# Clinical and genetic characteristics of 100 consecutive patients with Birt-Hogg-Dubé syndrome in Eastern Chinese region

**DOI:** 10.1186/s13023-024-03360-1

**Published:** 2024-09-19

**Authors:** Daiju Hu, Rui Wang, Jinli Liu, Xianmeng Chen, Xianliang Jiang, Jun Xiao, Jay H. Ryu, Xiaowen Hu

**Affiliations:** 1 Department of Pulmonary and Critical Care Medicine, Hefei, China; 2 Department of Dermatology, Hefei, China; 3 Center for Diagnosis and Management of Rare Diseases, Hefei, China; 4 Department of Thoracic Surgery, Hefei, China; 5 Department of Urology, Division of Life Sciences and Medicine, The First Affiliated Hospital of USTC, University of Science and Technology of China, Hefei, China; 6 WanNan Medical College, Wuhu, China; 7 Division of Pulmonary and Critical Care Medicine, Mayo Clinic, Rochester, MN USA

**Keywords:** Birt-Hogg-Dubé syndrome, *FLCN* mutation, Diagnostic criteria

## Abstract

**Background:**

Although an increasing number of patients with Birt-Hogg-Dubé syndrome (BHD) are being recognized in China, clinical and genetic characteristics are not well-defined. In addition, revised diagnostic criteria for the Chinese population was proposed in 2023, we aimed to explore their utility in clinical practice at a rare lung disease center.

**Methods:**

We retrospectively analyzed the data of 100 consecutive patients with BHD diagnosed according to the revised Chinese BHD criteria, encountered at the First Affiliated Hospital of University of Science and Technology of China from Jan 2017 to June 2023.

**Results:**

There were 100 patients (including 63 females) from 65 unrelated families in Eastern China, mostly Anhui Province. The common manifestations were pulmonary cysts (99%), pneumothorax (60%), and skin lesions (77%). Renal cancer and renal angiomyolipoma were detected in 5 patients each. 37% of patients had no family history of BHD. In total, 25 *FLCN* germline mutations were detected, including 6 novel mutations. In addition to hotspot mutation c.1285delC/dupC (17%), the most common mutations were c.1015 C > T (16%), c.1579_1580insA (14%), and exons 1–3 deletion (11%) in *FLCN*. Higher risk of pneumothorax was associated with exons 1–3 deletion mutation and c.1177-5_1177-3de1CTC compared to the hotspot mutation c.1285dupC (91% [95% CI: 0.31, 46.82, *p* = 0.015] and 67% [95% CI: 0.35, 71.9, *p* = 0.302] vs. 30%, respectively). The average delay in diagnosis was 7.6 years after initial symptoms. Chinese diagnostic criteria were mostly consistent with typical pulmonary presentations with supportive genetic evidence.

**Conclusion:**

In the Eastern Chinese region, patients with BHD present most commonly with pulmonary cysts associated with pneumothorax and skin lesions. However, low incidence of renal cancer along with unexpected renal angiomyolipoma was observed. Genotypic spectrum differed from that reported from other global regions, and genotype association of pneumothorax warrants further research. The revised Chinese criteria for BHD seem more appropriate in diagnosing BHD in Chinese patients.

**Supplementary Information:**

The online version contains supplementary material available at 10.1186/s13023-024-03360-1.

## Introduction

Birt-Hogg-Dubé syndrome (BHD) is a rare autosomal dominant disorder caused by germline mutations in the folliculin (*FLCN*) gene [1]. The main manifestations are multiple pulmonary cysts, fibrofolliculomas, recurrent pneumothorax, and renal cancers [2]. The *FLCN* gene, located on chromosome 17p11.2, was identified as a tumor suppressor gene in 2002 [3] and is responsible for BHD syndrome. To date, over 300 pathogenic variants have been reported worldwide, most of which are point mutations resulting in premature protein truncation [4]. In previous reports [5–8], *FLCN* gene mutations in BHD syndrome in China differed from those in Europe and the United States. For example, among 51 Chinese patients with suspected BHD, 27 had *FLCN* germline mutations including 14 novel mutations [8]. However, the limited number of cases in this study couldn’t reflect the whole profile of countrywide patients due to mostly from Northern China.

Although an increasing number of Chinese patients with BHD are being reported in recent years, failure in recognition and delay in diagnosis remains a challenge for patients with BHD. Recent reports suggest that Chinese patients with BHD manifest predominantly pulmonary abnormalities, with less frequent skin and kidney involvement [9]. Thus, BHD in the Caucasian population seems predominantly characterized by lung lesions combined with skin and renal malignancies, while the Asian patients manifest a lower incidence of skin lesions [10, 11].

The diagnostic criteria proposed by the European BHD Consortium in 2009 [12] which is mainly based on the presenting manifestations seen in Caucasian patients may not be appropriate for the Chinese population. Thus, a revised set of diagnostic criteria was proposed in 2023 by Chinese experts to improve the diagnosis of BHD (Table 1). This set of diagnostic criteria focuses firstly on the clinical presentation (multiple lung cysts, rash and renal cancer), then combined with genetic diagnostic criteria. Therefore, it is more in line with the low prevalence of skin and kidney lesions in Chinese patients with BHD, which would facilitate a timely diagnosis in this population.

**Table 1:** Diagnostic criteria for Birt-Hogg-Dubé syndrome (China) [10]

Diagnosis criteria
Clinical criteria Multiple lung cysts: diffuse lung cysts with no other apparent cause, mainly at the basal and mediastinum with or without spontaneous pneumothorax Rash: at least five fibrofolliculomas or trichodiscomas, mostly on face or neck Renal cancer: early onset (age < 50 years), or multifocal or bilateral renal cancer, or renal cancer of mixed chromophobe and oncocytic histology
Genetic criteria
Pathogenic *FLCN* germline mutation A first-degree relative with BHD

*Note* BHD: Birt-Hogg-Dubé Syndrome. For diagnosis of BHD, one clinical criterion plus genetic criteria or two clinical criteria are required

Herein, we retrospectively analyzed the data of 100 consecutive patients with BHD encountered in Eastern Chinese region to provide additional data pertaining to this rare disease, and to validate the new Chinese criteria for the diagnosis of BHD in clinical practice at a rare lung disease center.

## Subjects and methods

### Study population

Approval was obtained from the ethics committee of the First Affiliated Hospital of the University of Science and Technology of China in Anhui Province (reference number 2023-RE-290). All the patients diagnosed with BHD were recruited from inpatient wards or Rare Lung Disease Clinic - supported by a multidisciplinary team, from January 1, 2017 to June 30, 2023. Informed consents were obtained from all patients, except for one deceased patient who was exempt from the informed consent requirements. The diagnosis of BHD was based on the Chinese criteria proposed by Expert Consensus on the Diagnosis and Management of BHD in 2023 [13]. Clinical data of all patients including gender, age at diagnosis, smoking history, prior medical history (pneumothorax, skin lesion, and renal cancer), family history, imaging studies and genetic testing were reviewed (Table 2).

**Table 2:** Demographic and clinical characteristics of 100 patients with Birt‑Hogg‑Dubé syndrome

Characteristics	Cases with available data, *n*	Value, *n* (%)
Male	100	37 (37)
Age at examination-yr	100	45.3 ± 13.7 (15–81)
Average time to diagnosis-yr	100	7.6 ± 8.3
Smoking history	100	11 (11)
*FLCN* Genetic testing	86	85 (99)
Point mutation	85	73 (86)
Fragment deletion	85	12 (14)
Family history of pneumothorax	100	63 (63)
Cysts on chest CT	96	95 (99)
Pneumothorax	100	60 (60)
Skin manifestations	86	66 (77)
Cancer	76	5 (7)
AML	76	5 (7)

Age was presented as mean ± standard deviation (range) and categorical variables were presented as percentages

### Mutation analysis of the *FLCN* gene

Genomic DNA from 86 patients was extracted from peripheral blood leukocytes and assayed by polymerase chain reaction (PCR) and Sanger sequencing. Multiplex ligation-dependent probe amplification (MLPA) reactions carried out in accordance with the manufacturer’s guidelines. The PCR products were examined using an ABI 3130 Genetic Analyzer from Applied Biosystems. The data were analyzed with the Coffalyser software from MRC-Holland. The PCR amplification kit (Takara, China) was used to amplify the junction fragments adjacent to the deleted regions, using specially designed primers (F CTGAGGGACACCAAGCACTC, R TGGGAAAGATGTTAATGGCCTA). PCR products were separated by 2% agarose gel electrophoresis. Bidirectional Sanger sequencing was performed. *FLCN* mutations were numbered based on GenBank accession numbers NM_144997.7 according to the HGVS nomenclature guideline (http://www.hgvs.org/mutnomen).

### Statistical analysis

Statistical analyses were conducted using SPSS version 26.0. Continuous variables were presented as means and standard deviations, and then compared using independent sample t-tests. Categorical variables were presented as frequencies and percentages, and were analyzed using the chi-square test and Fisher’s exact test for comparison. A P-value below 0.05 was deemed statistically significant.

## Results

### Clinical features

The 100 patients were all of Han ethnicity and from 65 different families. Ninety-three cases were original citizens of Anhui Province, Eastern area of China. The remaining 7 patients were from Jiangsu, Shandong, Fujian and Sichuan Provinces in China, respectively. The average age of the patients at diagnosis was 45.3 years (ranging from 15 to 81 years old). There were 37 males with a male to female ratio of 1:1.7. Eleven patients had a smoking history, while 63 cases had a familial history of pneumothorax(Table 2).

**Fig. 1:**
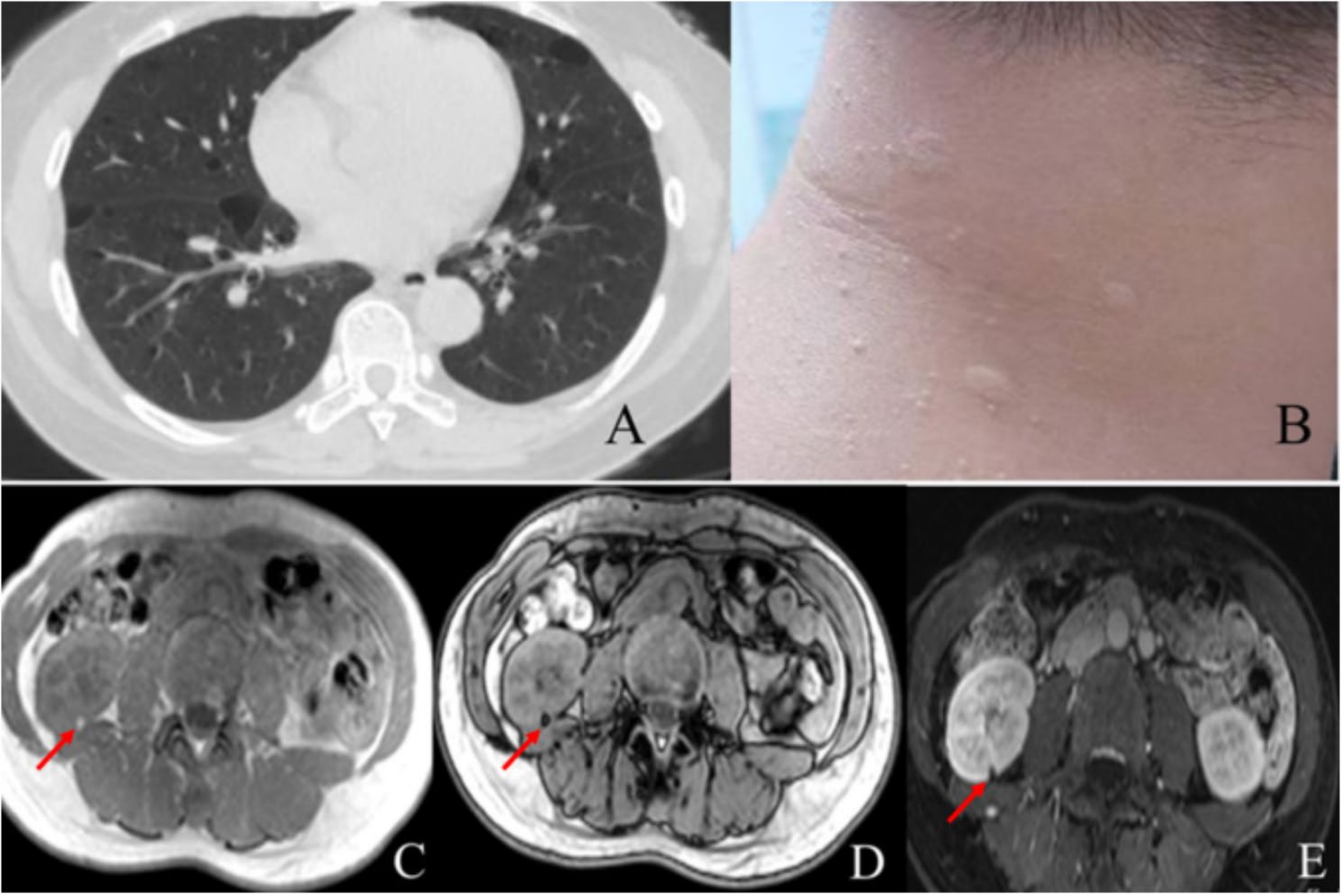
**A**:CT of patient 1 (44-year-old female) of family 8 shows multiple cysts distributed in the lower lung. **B**: Patient 1 (35-year-old male) of family 52 had fibrofolliculomas on the face and neck. **C**, **D**, **E**: Magnetic resonance imaging of the kidneys showing angiomyolipoma (patient 1 of family 12):C, focal abnormally high signal in the right kidney. **D**, decreased signal in the out of phase lesion suggesting adipose tissue. E, no enhancement on enhanced scan also suggests adipose tissue

Of the 100 patients with BHD,60% had a history of pneumothorax.Forty-four patients with pneumothorax underwent surgery (including video-assisted thoracoscopic biopsy, thoracotomy with biopsy, and mechanical pleurodesis).The lung pathology in all patients was consistent with pulmonary cysts/bullae. In addition, there was adenocarcinoma of the lung in three of them.Chest CT examination was performed in 96 patients, and lung cysts were detected in 95 of them (99%).The number of cysts were not assessed in 12 patients due to chest CT study having been performed at other hospitals and not available for current review, and 4 cases were confirmed by family investigation without chest CT examination. Review of chest CT imaging available in 84 patients demonstrated multiple pulmonary cystic lesions in all. The number of lung cysts were found to be less than 10 (6 cases), 10–20 (25 cases), and over 20 (52 cases), respectively. All of these patients had cystic lesions bilaterally, except for 1 case (F5-2).The cysts were in the lower lungs in 67 cases (81%) and showed diffuse distribution in 15 cases (18%).In addition, three patients had pulmonary malignant tumors, from families 6 and 11, respectively.The pathological types were adenocarcinoma in 2 cases and sclerosing alveolar cell tumor in the remaining one. (Fig. 1-A).

**Fig. 2:**
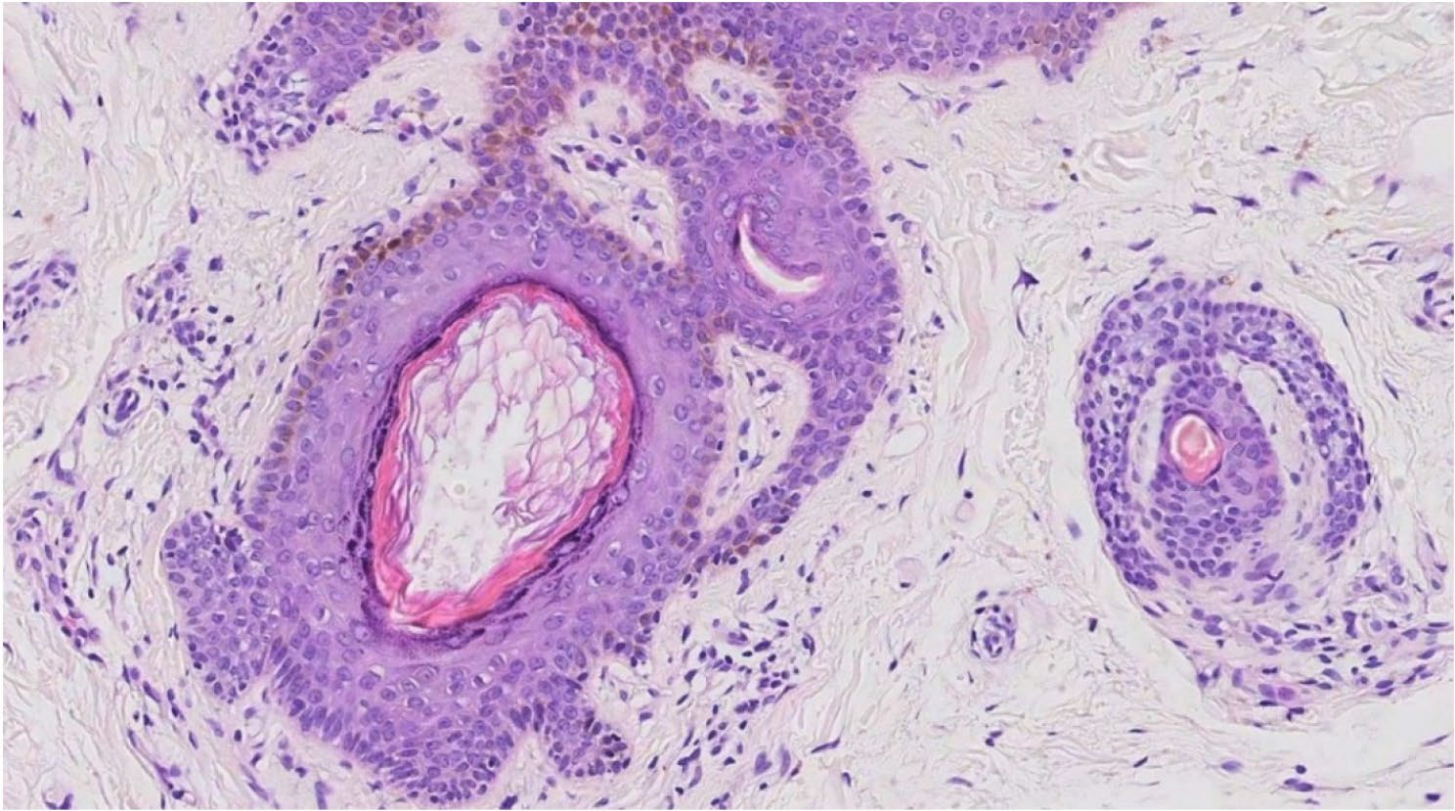
Skin biopsy of patient 74 (66-year-old male) of family 45: Hematoxylin and eosin staining of skin biopsy showing histologic features consistent with fibrofolliculoma (×200)

Eighty-six patients were examined by dermatologists and 66 patients (77%) were noted to have skin lesions. Histopathological confirmation of epidermoid cysts was seen in 6 patients, histopathological confirmation of fibrofolliculoma (FF)/trichodiscoma (TD) was achieved in 5 patients (Figs. 1-B and 2). The remaining patients were clinically diagnosed based on skin assessment as many individuals refused to undergo skin biopsy.

Renal imaging examination was performed in 78 patients, and 10 patients were found to have renal tumors (13%), including 5 cases of renal cancer and 5 cases of renal angiomyolipoma (AML). Three of the five patients diagnosed with renal cancer underwent nephrectomy. The histopathologic types were chromophobe, clear cell, and low-grade eosinophilic renal cell carcinoma. The remaining two patients with renal cancer were diagnosed on imaging without histopathologic confirmation. One received renal artery chemoembolization, and the remaining patient is still being monitored. Five patients with renal angiomyolipoma (AML) came from family 6, family 8, family 12 and family 14, respectively. Two patients with AML from family 8 were siblings. The characteristics and genetic results of the five AML patients in our cohort are shown in Supplement Table 2; Fig. 1-C, D,E.

### Germline mutation of the *FLCN* gene

**Fig. 3:**
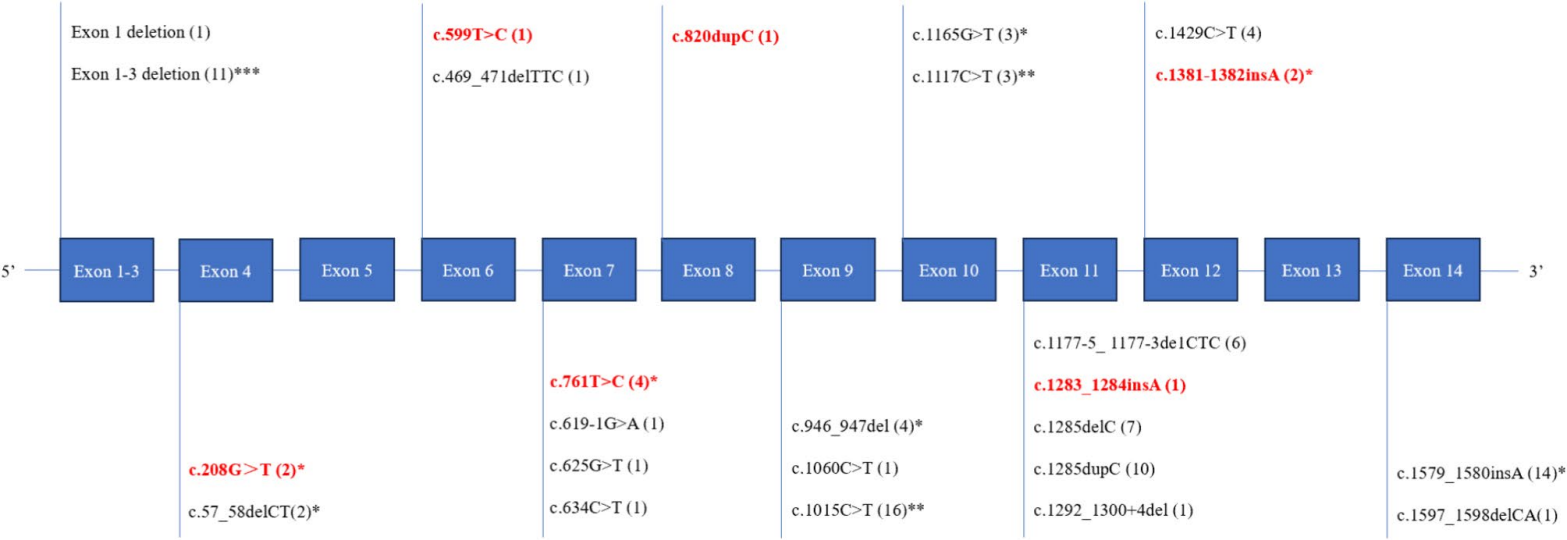
Mapping of *FLCN* variants for patients with Birt‑Hogg‑Dubé syndrome *25 *FLCN* mutations among 86 patients, including 6 novel mutations (marked in red). Numbers of patients shown within parentheses

Of the 100 patients with BHD, 85 cases were confirmed by genetic testing, 1 case diagnosed pathologically by skin biopsy, and the other 14 cases were diagnosed by clinical criteria alone. A total of 25 pathogenic gene mutations were detected. There were two significant gene fragment deletions (exon 1 and a heterozygous deletion spanning exons 1–3) and 23 point mutations detected.The 23 mutations included 9 frameshift mutations, 8 nonsense mutations, 2 splice site mutations, 2 missense mutations, 1 in-frame mutation, and 1 uncharacterized species (F59-1, exon11 c.1292_1300 + 4del). The most frequent mutations were c.1285dup/del C, c.1015 C> T, c.1579_1580insA, exon1-3 del and c.1177_5 1177_3de1CTC, respectively. In addition, 6 novel mutations were identified (Fig. 3).

### Genotype–phenotype correlations

Pulmonary cysts were identified across all gene variants, with no statistically significant differences observed between the various genotypes. However, spontaneous pneumothorax was prevalent in those with exon1-3 del (91%), c.1285delC (85%), c.1177_51177_3de1CTC (67%), c.1579_1580insA (50%), and c.1015 C> T (44%). Furthermore, the exon1-3 deletion had a significantly higher risk of pneumothorax (91%) compared to c.1285dupC gene (30%) (95% CI: 0.31, 46.82, *p* = 0.015). The corresponding mutation types in the five renal cancer patients were exon 7 c.1364–1365 ins T, exon 12 c.1429 C> T, exon 1–3 deletion, and exon 11 c.1177-5_1177-3delCTC, respectively. Two patients with exon 1–3 deletion from family 34 were found to have both renal cancer and renal cyst lesions. The types of genetic variants of the 5 patients with renal angiomyolipoma were completely different including exon 14, c.1579_1580insA, exon 10, c.1177-5_1177-3delCTC, exon 7, c.T761C, and exon 1–3 deletion.

In 66 patients with skin lesions, the gene mutation sites were c.1015 C> T (10 patients), c.1285dupC (10 patients), c.1285delC (6 patients), c.1177_5 1177_3de1CTC (5 patients), exon1-3 deletion (9 patients), and c.1579_1580insA (8 patients), respectively. No association was found between genetic mutation sites in patients with kidney findings and skin lesions.

**Fig. 4:**
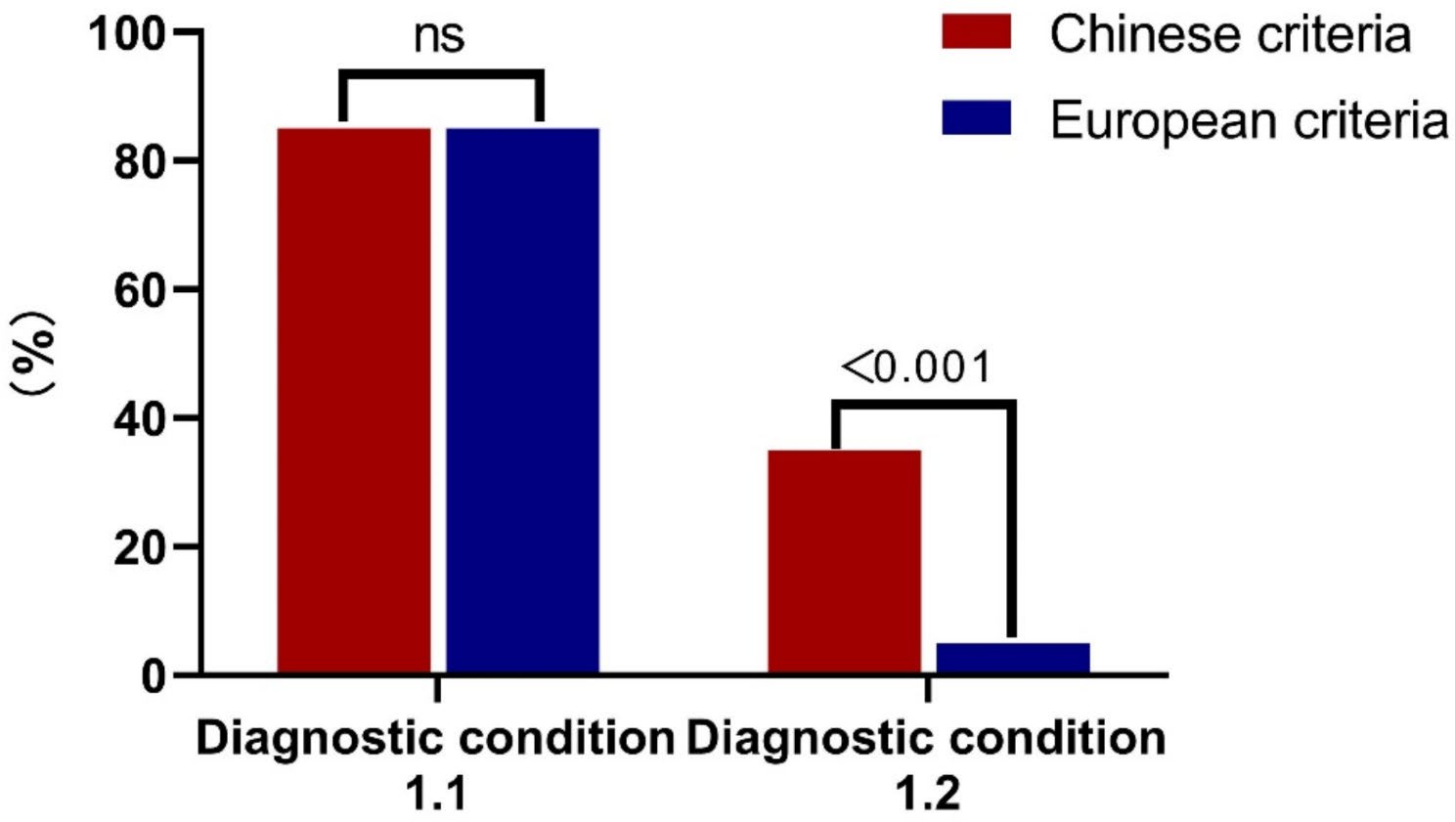
Comparison of two different sets of diagnostic criteria for 100 Chinese patients with Birt‑Hogg‑Dubé syndrome. *Note* BHD: Birt-Hogg-Dubé Syndrome; Diagnosis condition 1.1 Pathogenic *FLCN* germline mutation; Diagnosis condition 1.2: A first-degree relative with BHD for Chinese criteria and histologically confirmed skin lesions for European criteria

The average duration between the onset of symptoms and the diagnosis of BHD was 7.6 years. According to the European BHD diagnostic criteria, there were only 5 (6.4%) patients who met the major criterion of skin lesions, 85 (98.8%) cases met with major criterion of positive genetic testing. According to the revised Chinese BHD criteria, 95 lung lesions (99%), 5 kidney cancers (6.4%), and 5 (5.8%) skin lesions met the clinical diagnostic criteria, 85 (98.8%) pathogenic *FLCN* mutations met the genetic diagnostic criteria, and 35 (35%) had first-degree relatives with confirmed diagnosis. (Fig. 4; Table 3)

**Table 3:** Comparison of Chinese and European diagnostic criteria for 100 patients with birt hogg dubé syndrome

Diagnostic condition	1.1 (*n* = 86)	1.2 (*n* = 100)	2.1 (*n* = 96)	2.2 (*n* = 78)	2.3 (*n* = 86)
Chinese criteria	98.8%	35.0%^**#**^	99.0%	6.4%	5.8%*
European criteria	98.8%	5.8%*^$^	99.0%	6.4%	35.0%^**#&**^
*p*-value	NA	<0.001	NA	NA	<0.001

*Note* 1.1 Pathogenic *FLCN* germline mutation;1.2 At least five fibrofolliculomas or trichodiscomas / A first-degree relative with BHD (* Skin lesions and ^#^A first-degree relative with BHD); 2.1 Multiple lung cysts; 2.2 Renal cancer; 2.3 is similar to1.2. ^$^ 86 patients; ^&^100patients. NA: not available

## Discussion

BHD syndrome was first described in 1975 as a rare autosomal dominant disorder characterized by cutaneous fibrofolliculomas, pulmonary cysts, and renal tumors [14]. According to the BHD Foundation, over 600 families have been reported globally [4]. In the current report, we describe the clinical and genetic characteristics of 100 consecutive patients with BHD from Eastern China, which is the largest cohort reported from the Chinese population to date. Current study based on patients referred to a Rare Lung Disease Clinic with a multidisciplinary team of specialists, demonstrated that revised Chinese criteria can improve the diagnosis of BHD in China. The results of our study showed pulmonary cysts as the main manifestation with a high incidence of skin lesions, while the frequency of renal tumors was low. We also identified 5 patients with unexpected renal angiomyolipoma and 6 novel *FLCN* mutations. Furthermore, our study revealed that more than 90% of patients with exons 1–3 deletion experienced pneumothorax.

Pulmonary cysts are the most common presentation of BHD. Imaging findings of pulmonary cysts of variable size, irregular shape, and basal anterior distribution are considered important clues for the diagnosis of BHD [15]. Among Chinese patients with BHD, the most common manifestations were pulmonary including diffuse cysts and spontaneous pneumothorax. For example, lung cysts were noted in 92% of patients in prior report [16]. Due to the high prevalence of lung cysts in adult patients with BHD, spontaneous pneumothorax can also be a common presentation [17, 18]. In our study, almost all the patients had pulmonary cysts and 60% of them had a history of pneumothorax. This was consistent with BHD reported from other Asian countries [8]. Almost half of our patients underwent thoracic surgery for recurrent pneumothorax, which yielded pathologic result consistent with pulmonary cysts/bullae. In the setting of cystic lung disease, recurrent spontaneous pneumothorax in a non-smoker should alert the pathologist to the underlying lung pathology, which may be linked to *FLCN* gene mutation, in order to find further evidence of BHD.

In 2016, Furuya et al. reported 14 pulmonary neoplastic lesions in 7 patients with BHD, including adenocarcinoma in situ (*n* = 2), minimally invasive adenocarcinoma (*n* = 1), papillary adenocarcinoma (*n* = 1), micropapillary adenocarcinoma (*n* = 1), and atypical adenomatous hyperplasia (*n* = 8) [19]. We also found three cases of combined lung cancer, and all of them were pathologically suggestive of adenocarcinoma.Potential risk for colon cancer has also been reported in recent BHD studies [20, 21].However, none of these three patients underwent genetic profiling of lung cancer. Therefore, further studies clarifying the risk of malignant tumor in those with BHD seem warranted.

The incidence of characteristic skin lesions in BHD ranges from 75 to 90% in the Caucasian population [17, 20]. Prior studies had indicated that Asian patients have a notably lower incidence of skin lesions (30–48%) compared to Caucasian patients [22, 23]. However, our data showed that the incidence of skin lesions was 77%. This higher incidence may be explained by the input of experienced dermatology specialists within our multidisciplinary team, which improved the detection rate for skin lesions. As such, further investigation is needed to explore the prevalence of skin lesions in Asian patients with BHD, and it is important to encourage more patients with skin lesions to undergo skin biopsy to confirm the diagnosis.

Renal tumors occur in 25–35% of patients with BHD and are usually bilateral, multifocal, and slow-growing. Compared with the skin and lung manifestations of BHD, renal tumors are more important for the long-term prognosis of BHD patients.Thus, surveillance for early detection and diagnosis is essential for improving prognosis.Renal tumors associated with BHD consist predominantly of hybrid chromophobe/oncocytic tumors (67%), chromophobe renal cell tumors (23%), clear cell renal carcinoma (7%), and renal oncocytomas (3%) [24]. These pathological types are less common in the general population. Consequently, the possibility of BHD should be considered when the aforementioned pathological features are encountered. Previous studies indicate distinctive cytogenetic features in *FLCN* mutation related RCC compared to sporadic RCC [25].A study from Japan found that 34.8% of individuals carrying *FLCN* mutations aged over 40 had been diagnosed with renal cancers [16]. The occurrence of renal cancer in our cohort was exceptionally low compared to other reports from China. This could be explained by the limited follow-up duration. It’s noteworthy that 5 patients (6.4%) were detected with renal angiomyolipoma, which were only reported as individual cases. The incidence of AML in the general population is reported to be 0.1–0.22% [26]. Renal AMLs were found in 41% of patients diagnosed with sporadic LAM, and in 96% of individuals with TSC-LAM [27]. In 2012, Byrne et al. firstly described a 39-year-old woman diagnosed with BHD and a renal AML [28]. The reported clinical similarities between BHD and TSC may arise from the overlapping functions of FLCN (FNIP1 or FNIP2) and TSC (TSC1 or TSC2) proteins in the mTOR pathway, particularly in the assembly of the mTOR complex 1 [29]. Current guidelines recommend the use of sirolimus and other mTOR blockers in the treatment of AML, and therapeutic efficacy have been demonstrated [30, 31]. This strategy may also hold promise in the treatment of renal angiomyolipomas in patients with BHD.

*FLCN* mutation profiles have been reported with some variations in different populations. In the Caucasian populations, a cytosine insertion/deletion in a C8 tract in exon 11 is a mutation hotspot for BHD. Most BHD mutations are expected to result in truncation of the BHD protein, folliculin [32].In Japanese populations, the common mutations were c.1285dupC, c.1533_1536delGATG, and c.1347_1353dupCCACCCT [11]. By the end of 2021, there were 287 patients with BHD from 143 families reported in China. As reported in previous studies, the most frequent mutation was the single deletion, duplication of cytosine in codon 1285 of exon 11 (25%), following by the mutation of c.1579_1580ins in exon 14 (4.2%), and c.1015 C > T in exon 6 was the third most common mutation (3.3%) [14]. In total, 25 *FLCN* mutations were detected in the present study, and one-quarter of *FLCN* mutations were novel. Except for hotspot mutation c.1285delC/dupC, the other frequent mutations in our cohort were different from other areas in China. For example, the mutations percentage of c.1015 C > T, c.1579 and 1580insA were higher than that of previous report [9].

The relationship between gene mutation sites and clinical phenotypes has always been a focus of attention in various studies. A recent study from Germany found mutation c.924_926del to be associated with a 39% risk for pneumothorax, which increased to 60% for mutation c.1285dup, and 73% for mutation c. 1579_1580ins [33]. Our results showed that c.1285delC, c.1177_5 1177_3de1CTC and exon 1–3 deletion were associated with a high incidence of pneumothorax. In particular, the incidence of pneumothorax in exon 1–3 deletion patients was significantly higher than that in mutation hotspot c.1285dup patients reported in previous study [34]. Thus, BHD in Eastern China presented different genotypic characteristics from other areas and its association of pneumothorax phenotype warrants further research.

The delay in diagnosis remains a challenge both in China and in the rest of the world. The average time of diagnostic delay was 7.6 years in this study and almost 10 years in China overall [35]. In 2009, the European BHD Syndrome Consortium introduced the diagnostic criteria for BHD, which have since become widely utilized in clinical settings [12]. The European BHD diagnostic criteria suggest that a minimum of five fibrofolliculomas or trichodiscomas (with at least one confirmed histologically, occurring in adulthood) and the presence of a pathogenic *FLCN* germline mutation to be major criteria. Minor diagnostic criteria include multiple pulmonary cysts, renal cancer, and first-degree relatives diagnosed with BHD. Currently, Chinese patients with BHD have a lower frequency of positive family history and a low biopsy rate of skin lesions, plus low incidence of renal cancer. According to the European BHD diagnostic criteria, only few Chinese cases met the major criterion of skin lesions, which is not conductive to early diagnosis of BHD in China. Therefore, in 2023, a revised criterion for BHD proposed by China Alliance for the Rare Lung Disease and experts from the related disciplines in China [13]. Considering the clinical characteristics and realities of BHD in China, it optimized the diagnostic criteria by adding family history of first-degree relatives as a genetic criterion and adjusting skin lesion as a clinical criterion. In general, the diagnosis of hereditary diseases is usually based on the clinical phenotype and ultimately supported by the positive genetic mutation. Therefore, as a hereditary disease, BHD could also be characterized in this fashion. The Chinese diagnostic criteria summarize the clinical and genetic manifestations, which is more in line with the characteristics of hereditary diseases and is suitable for Chinese patients with low skin biopsy rate. Our study also confirmed the revised diagnostic criteria might be more applicable for Chinese patients with BHD (Table 3), the difference was statistically significant when skin pathology was used as a main diagnostic criterion (95%CI: 3.48, 27.85, *p* < 0.001).

Nevertheless, we acknowledge the limits of the present study, such as its retrospective design and the significant number of patients who did not undergo skin lesion biopsy. In addition, there may have been selection bias associated with most of our patients being diagnosed through referral to the Rare Lung Disease Clinic. A more accurate assessment of this issue could be performed in a multi-center prospective study using a standard protocol in family screening and systemic follow-up evaluation for this rare disease. Nonetheless, considering the rarity of BHD disease, we have collected the largest number of cases up to date. Therefore, our findings offer valuable insights to improve the understanding and diagnosis of this rare disease in China.

## Conclusion

In Eastern China, patients with BHD commonly exhibit pulmonary cysts, pneumothorax, and skin lesions. However, rather low incidence of kidney cancer along with unexpected renal angiomyolipomas were noted in this cohort. Our cohort manifested different genotypic characteristics from other populations, and the association of pneumothorax with genotypes warrant further investigations. The revised Chinese criteria might be more useful in diagnosing BHD in the Chinese population, compared the traditional criteria.

## Electronic supplementary material

Below is the link to the electronic supplementary material.

Supplement Table 1. Genetic Characterization and Clinical Phenotypes of 100 patients with Birt‑Hogg‑Dubé syndrome

Supplement Table 2.Clinical Characteristics of the five patients with Birt‑Hogg‑Dubé syndrome and Renal AML

## Data Availability

All data generated or analyzed during this study are included in this published article and its supplementary information files.
