# Supplementary material for: Clinical and genetic characteristics of 100 consecutive patients with Birt-Hogg-Dubé syndrome in Eastern Chinese region
Source: Orphanet J Rare Dis. 2024 Sep 19;19:348. doi: 10.1186/s13023-024-03360-1 (PMC11414263; doi:10.1186/s13023-024-03360-1)
Supplement: Supplementary file 1 — Supplement Table 1. Genetic Characterization and Clinical Phenotypes of 100 patients with Birt‑Hogg‑Dubé syndrome [file 13023_2024_3360_MOESM1_ESM.docx]

Supplement Table 1. Genetic Characterization and Clinical Phenotypes of 100 patients with Birt‑Hogg‑Dubé syndrome

|  | **Mutation analysis** |  | **Phenotype in family members** |  |  |  |  |  |  |
| --- | --- | --- | --- | --- | --- | --- | --- | --- | --- |
| Family ID | Exon | Nucleotide change | No. with  lung cysts | No. with  pneumo-thorax | Other pulmonary lesions | No.with  FF/TD | No.with multiple skin-colored papules | No. with  Renal tumours | No. with  kidney cyst |
| 1 | 10 | c.1165G>T | 2(2) | 2(2) | 0 | 0(2) | 1(2) | 0(2) | 0(2) |
| 2 | 4 | c.208G>Tp.Glu70* | 2(2) | 0(2) | 0 | 0(2) | 1(2) | 0(2) | 0(2) |
| 3 | 9 | rs8065832,+6bp,Re:CTT pure c.1015C>T p.Gln339* | 3(3) | 1(3) |  | 0(3) | 1(3) | 0(3) | 0(3) |
| 4 | 6 | rs1736219,-14bp Re:C)--CT heterozygous c.469_471delTTC p.Phe157del | 1(1) | 1(1) | 0 | 0 | 1(1) | 0 | 0 |
| 5 | 9 | rs8065832,+6bp,Re:C)--TTT pure c.1015C>T p.Gln339* | 2(2) | 1(2) | 0 | 0 | 1(2) | 0 | 1(2) |
| 6 | 14 | c.1579_1580insA p.Arg527Glnfs*75 | 7(7) | 4(7) | lung cancer1(7) | 0 | 5(7) | AML 1(7) | 1(7) |
| 7 | 14 | c.1597_1598delCA p.Gln533Glufs*68 | 1 | 0 | 0 | 0 | 0 | 0 |  |
| 8 | 11 | c.1177-5_1177-3delCTC | 2(2) | 0 | 0 | 0 | 2(2) | AML 2(2) | 0 |
| 9 | 11 | c.1285dupC p.His429Profs*27 | 1 | 1 | 0 | 0 | 1 | 0 | 0 |
| 10 | 11 | c.1177-5_1177-3delCTC | 1 | 1 | 0 | 0 | 0 | 0 | 0 |
| 11 | 11 | c.1285del | 1(2) | 1(2) | Lung cancer1(2) | 1(2) | 0 | 0 | 1(2) |
| 12 | 7 | c.T761C | 2(2) | 2(2) | 0 |  | 2(2) | AML1(2) | 1(2) |
| 13 | 1-3 | Exon 1, Exon 2 and Exon 3 deletion | 1(1) | 1 | 0 | 0 | 0 | 0 |  |
| 14 | 1-3 | Exon 1, Exon 2 and Exon 3 deletion | 4(4) | 4 | 0 | 1 | 2 | 0 | 2(3) |
| 15 | 1-3 | Exon 1, Exon 2 and Exon 3 deletion | 1(1) | 1 | 0 | NA | NA | 0 | 0 |
| 16 | 10 | c.1165G>T | 1(1) | 1 | 0 | 0 | 0 | 0 |  |
| 17 | 11 | c.1285dup | 1(1) | 0 | 0 | 0 | 1(1) | 0 | 0 |
| 18 | 7 | c.761T>C | 1(1) | 1 | 0 | 0 | 0 | NA | NA |
| 19 | 11 | c.1285dup | 1(1) | 0 | 0 | 0 | 1 | 1 | 0 |
| 20 | 11 | c.1285del | 2(2) | 2 | 0 | 0 | 2 | 0 | 0 |
| 21 | 7 | c.1381-1382insA | 2(2) | 0 | 0 | 0 | 1(2) | Renal  cancer 1(2) | 0 |
| 22 | 9,6 | exon9 c.1062+6C>T、exon6 c.397-14C>T | 1(1) | 1 | 0 | 1 | 0 | 0 | 0 |
| 23 | 9 | c.1015C>T | 1(2) | 1(2) | 0 | 0 | 1(2) | 0 | 0 |
| 24 | 9 | c.946_947 del | 1(1) | 1(1) | 0 | 0 | 1(1) | 0 | 0 |
| 25 | 9 | c.1015C>T p.Gln339Ter | 1(1) | 0 | 0 | 0 | 1(1) | 0 | 0 |
| 26 | 7 | c.634C>T | 1(1) | 1(1) | 0 | 0 | 1(1) | 0 | 0 |
| 27 | 9 | c.761T>C | 1(1) | 0 | 0 | 0 | 1(1) | 0 | 1(1) |
| 28 | 11 | c.1285del | 1(1) | 1 | 0 | 0 | 1(1) | 0 | 0 |
| 29 | 12 | c.1429C>T | 2(2) | 0 | 0 | 0 | 1(2) | Renal cancer 1(2) | 0 |
| 30 | 11 | c.1283_1284insA | 1(1) | 1(1) | 0 | 0 | 1(1) | 0 | 0 |
| 31 | 11 | c.1285dupC | 2(2) | 1 | 0 | 0 | 2(2) | 0 | 0 |
| 32 | 14 | c.1579-1580insA | 1(3) | 1 | 0 | 0 | 1(3) | 0 | 0 |
| 33 | 14 | c.1579-1580insA | 1(1) | 1 | 0 | 0 | 1(1) | 0 | 0 |
| 34 | 1-3 | Heterozygous deletion mutations in exons 1-3 | 4(4) | 3 | 0 | 0 | 3(4) | Renal  cancer（4） | 2(2) |
| 35 | 11 | c.1285del | 1(1) | 1 | 0 | 0 | 1(1) | 0 | 0 |
| 36 | 1 | Exon 1 deletion | 1(1) | 0 | 0 | 0 | 1(1) | NA |  |
| 37 | 9 | c.1060C>T | 1(1) | 1 | 0 | 0 | 1(1) | 0 |  |
| 38 | 7 | c.619-1G>A | 1(1) | 1 | 0 | 0 | 1(1) | NA |  |
| 39 | 11 | c.1285dup | 1(1) | 1 | 0 | 0 | 1(1) | 0 | 1(1) |
| 40 | 1-3 | Exons 1-3 deletion | 1(1) | 1 | 0 | 0 | 1(1) | 0 | 1(1) |
| 41 | 6 | c.599T>C | 1(1) | 1 | 0 | 1 | 0 | 0 | 1(1) |
| 42 | 9 | c.1015C>T | 1(1) | 1 | 0 | 0 | 1(1) | 0 | 0 |
| 43 | 9 | c.946_947del: p.S316fs | 1(1) | 1 | 0 | 0 | 1(1) | 0 | 1(1) |
| 44 | 11 | c.1285dupC | 2(2) | 0 | 0 | 0 | 2(2) | 0 | 0 |
| 45 | 9 | c.946-947del: p.S316fs | 2(2) | 2 | 0 | 1 | NA | Adrenal adenoma1(2) | 0 |
| 46 | 11 | c.1285dupC | 1(1) | 0 | 0 | 0 | 1(1) | NA | 0 |
| 47 | 12 | c.1429C>T | 1(1) | 0 | 0 | 0 | 1(1) | 0 | 0 |
| 48 | 11 | c.1177-5_1177-3delCTC | 1(1) | 0 | 0 | 0 | 1(1) | 0 | 0 |
| 49 | 11 | c.1177-5_1177-3delCTC | 1(1) | 0 | 0 | 0 | 1(1) | 0 | 0 |
| 50 | 7 | c.625G>T | 1(1) | 0 | 0 | 0 | 1(1) | NA | 0 |
| 51 | 12 | c.1429C>T | 1(1) | 0 | 0 | 0 | 1(1) | 0 | 0 |
| 52 | 9 | c.1015C>T | 1(1) | 1 | 0 | 0 | 1(1) | 0 | 0 |
| 53 | 14 | c.1579_1580insA | 2(2) | 1 | 0 | 0 | 1(2) | NA | 0 |
| 54 | 9 | c.1015C>T | 1(1) | 1 | 0 | 0 | 1(1) | NA | 0 |
| 55 | 10 | c.1117C>T | 3(3) | 3 | 0 | 0 | 1(3) | 0 | 1(3) |
| 56 | 11 | c.1285delC | 1(1) | 1 | 0 | 0 | 1(1) | 0 | 0 |
| 57 | 9 | c.1015C>T | 1(1) | 0 | 0 | 0 | 1(1) | 0 | 0 |
| 58 | 11 | c.1285dupC | 1(1) | 0 | 0 | 0 | 1(1) | 0 | 0 |
| 59 | 11 | c.1292_1300+4del | 1(1) | 0 | 0 | 0 | 1(1) | 0 | 0 |
| 60 | 9 | c.C1015T/P.Q339X | 1(1) | 0 | 0 | 0 | 1(1) | 0 | 0 |
| 61 | 11 | c.1177-5_1177-3delCTC | 1(1) | 0 | 0 |  | 1(1) | Renal  cancer 1(1) | 0 |
| 62 | 4 | c.57_58delCT | 2(2) | 1 | 0 | 0 | 0 | 0 | 1(2) |
| 63 | 8 | c.820dupC | 1(1) | 1 | Tuberculosis 1(1) | 0 | 1(1) | 0 | 0 |
| 64 | 9 | c.C1015T/P.Q339X | 2(2) | 0 | Tuberculosis 1(2) | 0 | 1(2) | 0 | 1(1) |
| 65 | 9 | c.C1015T/P.Q339X | 1(1) | 0 | 0 | NA | NA | NA | NA |

Note: FF：fibrofolliculomas; TD:trichodiscomas; AML: angiomyolipoma; NA:Not Available.
