# Supplementary material for: Clinical and genetic characteristics of 100 consecutive patients with Birt-Hogg-Dubé syndrome in Eastern Chinese region
Source: Orphanet J Rare Dis. 2024 Sep 19;19:348. doi: 10.1186/s13023-024-03360-1 (PMC11414263; doi:10.1186/s13023-024-03360-1)
Supplement: Supplementary file 2 — Supplement Table 2.Clinical Characteristics of the five patients with Birt‑Hogg‑Dubé syndrome and Renal AML [file 13023_2024_3360_MOESM2_ESM.docx]

Supplement Table 2.Clinical Characteristics of the five patients with Birt‑Hogg‑Dubé syndrome and Renal AML

| Patient | Age, yr  /Gender | Clinical Presentation | *FLCN* gene  mutation | AML Diagnosis | AML Management | Follow-up information |
| --- | --- | --- | --- | --- | --- | --- |
| 1 | 54/F | Lung cysts, PTX,  Skin lesions | c.1579_1580insA | MRI  histopathology | Surgical operation | Stable  at 18 months |
| 2 | 45/M | Lung cysts,  Skin lesions | c.1177-5_1177-3delCTC | BUS | Observation | NA |
| 3 | 54/F | Lung cysts,  Skin lesions | c.1177-5_1177-3delCTC | Enhanced CT | Observation | Stable  at 16 months |
| 4  5 | 44/F  56/F | Lung cysts,  Skin lesions  Lung cysts, PTX,  Skin lesions | c.T761C  Exon 1-3 del | MRI  BUS | Observation  Observation | Stable  at 6 months  Stable  at 6 months |

Note: AML: angiomyolipoma; PTX: pneumothorax; MRI: magnetic resonance imaging; BUS: B-ultrasound.
